# Characteristics of alanine racemase in *Lactobacillus sakei* ZH‐2 strain

**DOI:** 10.1002/fsn3.3452

**Published:** 2023-06-01

**Authors:** Makoto Kanauchi, Natsuki Matsumoto

**Affiliations:** ^1^ School of Food, Agricultural and Environmental Sciences Miyagi University Sendai Japan; ^2^ Toasu Co., Ltd. Toyokawa Japan

**Keywords:** alanine racemase, enzyme, *Lactobacillus sakei*

## Abstract

Some d‐amino acid functions for food production are widely known: d‐alanine improves sensory evaluations of *sake*, beer, and fermented foods. Therefore, for the application of d‐amino acids, alanine racemase (ALRase) in *Lactobacillus sakei* ZH‐2, which has strong racemization, was analyzed using molecular biological methods. It had been hypothesized that ALRase coding DNA, *alr*, in ZH‐2 strain differs from those of other *Lactobacillus sakei* strains. However, complete genome sequencing by the National Center for Biotechnology (NCBI) revealed the amino acid sequence of *alr* in ZH‐2 strain to have homology of 99.4% similarity with the *alr* in *Lactobacillus sakei* 23K strain. However, it is considered that the sequence of *alr* was a unique amino acid sequence in the lactic acid bacteria group. DNA “*alr*” of ZH‐2 strain has a 1140 bp DNA base with 41 kDa molecular mass. Its molecular mass was inferred as approximately 38.0 kDa using SDS‐PAGE. Its optimum conditions are pH 9.0 at 30–40°C, showing stability at pH 9.0–10.0 and 4–40°C. Its cofactor is pyridoxal phosphate. Its activity is activated more by copper and zinc ions than by the lack of a metal ion. Additionally, its *K*_m_ is 1.32 × 10^−3^ (mol), with *V*_max_ of 4.27 × 10^−5^ (μmol^−1^ min^−1^). ALRase reacted against alanine most strongly in other substrates such as amino acids. The enzyme against serine was found to have 40% activity against alanine. The enzyme converted up to 54.5% of d‐alanine from l‐alanine ZH‐2 strain.

## Graphical abstract

For the application of d‐amino acids, alanine racemase (ALRase) in *Lactobacillus sakei* ZH‐2 was a unique amino acid sequence in the lactic acid bacteria group. Its molecular mass was inferred as approximately 38.0 kDa using SDS‐PAGE. Its optimum conditions are pH 9.0 at 30–40°C, showing stability at pH 9.0–10.0 and 4–40°C.

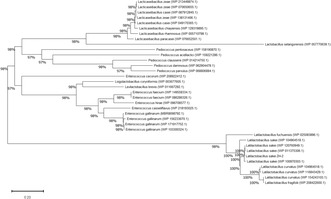

## 1 INTRODUCTION

Proteins are composed only of l‐amino acids: d‐amino acids are not amino acids for composing proteins. Furthermore, d‐amino acid amounts in nature are small. They are found in microorganisms, plants, fish, and animal bodies. Once, d‐amino acids were thought to have no role in human metabolism. For that reason, few studies examined d‐amino acids. Nevertheless, details of d‐amino acids in cells have been elucidated using several recently developed analytical technologies. Some researchers have reported the following roles of d‐amino acids in human metabolism and healthy function: d‐amino acid is present in the horny cell layer in human skin, which has a relation to skin aging (Gineyts et al., 2000). Moreover, d‐serine improves integration disorder syndrome because of its role in regulating N‐methyl‐D‐aspartate receptors in the human brain (Tsai et al., 1998). Moreover, d‐amino acids are broadly applicable to functional foods intended for health improvement because they enhance the intestinal environment. Large amounts of d‐amino acids have been found in fermented foods such as cheese (Bruckner & Hausch, 1989), beer (Bruckner & Hausch, 1989), *sake* (Gogami et al., 2011), wine (Erbe & Bruckner, 1998), and vinegar (Mutaguchi et al., 2013), which have more amino acids than fresh foods have (Marcone et al., 2020). Actually, d‐amino acids have stronger tastes than l‐amino acids. *Sake* containing d‐amino acids such as d‐alanine, d‐aspartic acid, and d‐glutamic acid is evaluated particularly highly in terms of sensory evaluation (Okada et al., 2013). Also, d‐amino acids have been produced by microorganisms during fermentation processes taking place in cheese, bread, and vinegar. Such fermented foods, therefore, have strong umami or sweet tastes. During vinegar production, main fermenting microorganisms such as *Acetobacter* spp. and lactic acid bacteria are used to produce d‐amino acids to enhance taste (Mutaguchi et al., 2013).

Reportedly, d‐amino acids in *sake*, which are produced by lactic acid bacteria using *Kimoto sake* seed mash brewing, depend on the type or species of lactic acid bacteria used for *sake moto* brewing (Kobayashi, 2019). For example, *Lactobacillus sakei* NBRC 15893 converts d‐alanine, d‐glutamic acid, and d‐aspartic acid using racemic enzymes (Kobayashi, 2019). The varieties and concentrations of racemized amino acids differ in the fermented solution because each microorganism's lactic acid bacteria have a varietal amino acid racemase. Therefore, for application to foods, many researchers should investigate varietal amino acid racemases from many lactic acid bacteria.

Matsumoto and Kanauchi (2019) reported that d‐amino acid assay developed rapidly and comprehensively using d‐amino acid oxidase and lactic acid bacteria *Lactobacillus sakei*. The lactic acid bacteria were isolated for tasty *sake* brewing. Reportedly, the isolated *Lactobacillus sakei* ZH‐2 strain had high racemization activity, with 14% conversion. The curd enzyme racemized l‐alanine and l‐serine. The findings indicate racemase as one kind of alanine racemase (ALRase). The produced d‐alanine improves *sake* sensory evaluation (Okada et al., 2013). Moreover, d‐amino acids improve the sensory evaluations of beer, wine, and several seasonings. During acid production, enzyme preparation for d‐amino acid racemase addition might be undertaken to improve their taste and other characteristics. Moreover, d‐serine, which facilitates human brain regulation, is a major gliotransmitter in mammalian central nervous systems (Hashimoto & Oka, 1997; Wolosker et al., 2008).

Applications of d‐amino acids have broad potential for producing *sake*, beer, wine, medicines, and healthy functional foods. Nevertheless, the racemase in ZH‐2, which converts a high level of d‐amino acid in a medium, has not been investigated for its characteristics, optimum conditions, or coding DNA base sequencing. As described herein, the enzyme was expressed by recombinant *Escherichia coli*. Recombinant racemase was investigated for its enzymic characteristics because it was intended for the application of racemase in *Lactobacillus sakei* ZH‐2 during food production, *sake* brewing, and beer brewing.

## 2 MATERIALS AND METHODS

### 2.1 Materials and chemicals

Chemicals for which no vendor is listed herein were purchased from Fujifilm Wako Pure Chemical Corp.

### 2.2 Cultivation of *Lactobacillus sakei* ZH‐2 strains

*Lactobacillus sakei* ZH‐2 strains were cultivated in GYP‐pantothenate medium comprising 1.0% glucose, 1.0% yeast extract (#551‐01310‐8; Kyokuto Pharmaceutical Industrial Co., Ltd.), 1.0% peptone (#551‐01010‐7; Kyokuto Pharmaceutical Industrial Co., Ltd.), 1.0% Tween 80 solution, and 0.5% mineral solution (containing 40 mg MgSO_4_·7H_2_O, 2 mg MnSO_4_·4H_2_O, 2 mg FeSO_4_·7H_2_O, 2 mg NaCl in 1 mL water, 3.0 μg/L calcium pantothenate) (#039‐14162; Fujifilm Wako Pure Chemical Corp.) at 30°C for 24 h (Matsumoto & Kanauchi, 2019).

### 2.3 Extraction of genomic DNA from *Lactobacillus sakei* ZH‐2

Genomic DNA was extracted according to the published procedures (Ausubel et al., 2003).

### 2.4 Amplification of *alr* coding ALRase

The *alr*, alanine racemase (ALRase) coding DNA, was amplified using a thermal cycler (T100; Bio‐Rad Laboratories Inc.) with Takara Ex Taq (#RR001A; Takara Bio Inc.) primers of two kinds (F‐ALR; 5′‐ATCTTCATCAGCCTCCGAAATC‐3′, R‐ALR; 5′‐ATGGTGTGGTT CACGTCTCGC T‐3′) with the genomic DNA extract solution as the template solution.

### 2.5 Inserting the *alr* to pMD20 T‐Vector and its transformation to competent cells

Ligation of insert DNA and vector transformation of host cells were conducted using the reported methods (Ausubel et al., 2003). Purified *alr* (120 ng) was ligated to pMD20 T‐Vector (20 ng, 2739 bp, 3270; Takara Bio Inc.) using Ligation Mix (Mighty Mix, #6023; Takara Bio Inc.) at 16°C for 30 min. Insertion of *alr* to pMD20 T‐Vector (pMD20‐alr) was transformed to *Escherichia coli* DH5α competent cells (#310‐06236; Nippon Gene Co., Ltd., Tokyo, Japan) using heat shock method at 42°C for 45–60 s.

### 2.6 Cultivation of transformation to competent cells and extraction ALRase from *Escherichia coli* DH5α‐pMD20‐*alr*

*Escherichia coli* DH5α‐pMD20‐*alr* were cultivated in 10 mL of LB medium with 0.1 M IPTG (isopropyl‐β‐D‐thiogalactoside). After cell cultivation, the cell mass was washed using 0.9% sodium chloride. After the cell mass was gathered by centrifugation at 20,400 *g* for 5 min at 4°C, the cell mass was resuspended in 0.5 mL of cell digestion buffer (50 mM Tris (trishydroxymethylaminomethane)‐hydrogen chloride (HCl); pH 8.0, 150 mM sodium chloride, 1 mM dithiothreitol). Then, 0.5 mL of lysozyme solution (containing 0.4 mg/mL) was poured as the final concentration. The mixture was incubated at 4°C for 30 min. After centrifugation, the supernatant was a crude enzyme solution.

### 2.7 Assaying ALRase activity

Using the modified method described by Matsumoto and Kanauchi (2019), the ALRase activity was assayed. Enzyme (50 μL) was added to 850 μL of 10 mM l‐alanine solution in 50 mM *N*‐cyclohexyl‐2‐aminoethanesulfonic acid (CHES, pH 9.0)‐NaOH (sodium hydroxide) buffer containing 10 mM pyridoxal 5′‐phosphate (PLP). Then, it was reacted at 37°C for 10 min. After the reaction, 50 μL of 100 mM hydrochloric acid solution was added to the reaction mixture to stop the reaction. It was then left to stand for 15 min at 37°C. After 50 μL of 100 mM sodium hydroxide solution was added to the reaction mixture to neutralize it, the mixture was centrifuged at 20,400 *g* for 5 min at 4°C. The supernatant (50 μL) and the amino acid oxidase solution (150 μL, 50 mM CHES‐NaOH buffer, pH 9.0, containing 0.6 μmol min^−1^ mL^−1^ d‐amino acid oxidase (DAO); 25 μmol min^−1^ mL^−1^ lactate dehydrogenase (LDH), and 0.2 mM nicotinamide adenine dinucleotide, reduced (NADH)) were poured to a 96‐well microplate (#1860‐096; AGC Techno Glass Co., Ltd.). The microplate absorbance was measured at 340 nm using a microplate reader (Multiskan FC; Thermo Fisher Scientific Inc.) after incubation at 37°C for 10 min. As Blank 1, assaying, phosphate buffer (50 mM, pH 6.0) instead of 50 μL of crude enzyme solution was added to 850 μL of 10 mM l‐alanine solution in 50 mM CHES buffer containing 10 mM PLP. Then, after it was reacted, 50 μL of crude enzyme added to 850 μL of CHES buffer containing PLP without l‐alanine was reacted as ‘Blank 2’ assaying to exclude effects of contaminating enzyme as LDH. After the reaction and centrifugation, the reaction solution was stopped. The solution was neutralized and centrifuged. All blank solutions (50 μL) and DAO‐LDH solution were poured into a 96‐well microplate. The absorbance was measured at 340 nm using a microplate reader after incubation at 37°C for 10 min. The ΔA_340_ was calculated according to the following formula.

(1) $$\begin{array}{c} \Delta A_{340}=\left[\left(A_{340}\;\text{of main sample assaying}\right)-\left(A_{340}\;\text{of Blank}\;1\;\text{assaying}\right)\right]\;\\ \;-\left[\left(A_{340}\;\text{of Blank}\;2\;\text{assaying}\right)-\left(A_{340}\;\text{of Blank}\;1\;\text{assaying}\right)\right] \end{array}$$

A standard curve used 0–1.0 mM d‐alanine dissolved in 50 mM CHES‐NaOH buffer. Both ΔA_340_ and the concentrations of d‐alanine are shown on the graph.

### 2.8 Assaying protein concentration

Protein was assayed using Bradford method (Bradford, 1976). The standard curve was produced using bovine serum albumin (#A2153; Sigma‐Aldrich Corp.).

### 2.9 DNA sequencing

The nucleotide base sequence of the *alr* was found using a DNA auto‐sequencer (ABI 3130; Applied Biosystems) with a kit (big Dye Terminator v 3.1 Cycle Sequencing; Applied Biosystems) according to the manufacturer's recommended protocols.

### 2.10 Homology analysis

The homology characteristics of each sequence were assessed using a Basic Local Alignment Search Tool (BLAST; NCBI, 2022).

### 2.11 Phylogenetic tree analysis of alanine racemase

Data of the nucleotide base sequences of 30 strains of genes of lactic acid bacteria were selected from the homology analysis by BLAST (NCBI, 2022), they were analyzed using neighbor‐joining method with software 11 (Molecular Evolutionary Genetics Analysis; Pennsylvania State University, 2022).

### 2.12 Preparation of pCold‐*alr* plasmid vector for high‐level expression recombinant alanine racemase (rALRase) by transformation of *Escherichia coli* LB21‐pCold‐*alr*

The ALRase coding DNA *alr* was re‐amplified using KOD DNA polymerase (#KOD‐101; Toyobo Co., Ltd.) and the plasmid DNA, with pMD20‐*alr as* the DNA template solution, which was extracted from *Escherichia coli* DH5α‐pMD20‐*alr* by Minipreps of the plasmid DNA method (Kobayashi et al., 2015). The following primers were used: forward primer F‐ALR 5′‐GCACGCATATGACAGTCGGTTA CTTACGAC‐3′; reverse primer R‐ALR 5′‐GCCGCGGATCCTTAGTGTTCATTTAATC CG‐3′. Amplified ALRase coding DNA (*alr*) was digested NdeI (#319‐01142; Nippon Gene Co., Ltd.) and BamHI (#315‐00061; Nippon Gene Co., Ltd.) at 37°C. The plasmid DNA as pCold I DNA (#3361; Takara Bio Inc.) was digested by NdeI and BamHI; then it was de‐phosphatized using CIAP (#2250; Takara Bio Inc.). Their DNAs were run using electrophoresis method. Their band was gel extracted from agarose gel using a kit (GEL/PCR Purification Mini kit). Purified alr (120 ng) was ligated to pCold I DNA (20 ng) using Ligation Mix (Mighty Mix, #6023; Takara Bio Inc.) at 16°C for 30 min. Insertion of *alr* to pCold‐*alr* was transformed to competent cell of *Escherichia coli* BL21 competent cells (#318‐06531; Nippon Gene Co., Ltd.) using heat shock method at 42°C for 45–60 s.

### 2.13 High‐level expression of recombinant alanine racemase (rALRase) by *Escherichia coli* LB21‐pCold‐*alr*

After the transformation of *Escherichia coli* LB21, SOC medium (100 μL) was added to the transformation cell mass. Then, they were incubated at 37°C for 60 min. After the cells were mass inoculated to LB plate medium (containing 100 μg/mL ampicillin), the medium was cultivated at 37°C for 16 h. Cells from each colony were inoculated to 10 mL of LB medium containing 100 μg/mL ampicillin using the medium cultivated until O.D._600_ (= 0.5). They were incubated and re‐cultivated in LB plate medium (containing 0.1 mM IPTG). The colony having the highest ALRase activity was selected as *Escherichia coli* LB21‐pCold‐*alr*.

### 2.14 Purification of rALRase from *Escherichia coli* LB21‐pCold‐*alr* by the affinity column for poly‐histidine affinity tag

rALRase extracted from *Escherichia coli* LB21‐pCold‐*alr* was purified using a HisLink Spin Protein Purification System (#V3680; Promega Corp.) according to the recommended protocol. To remove poly‐histidine, rALRase was treated with Factor Xa Protease (1 μL/mL, #P8010S; New England Biolabs Inc.). Then, Factor Xa was removed using a Factor Xa Cleavage Capture Kit (69037‐3; Merck, Darmstadt, B.R.D.). *Escherichia coli* BL21 competent cells were used to prepare negative control extraction.

### 2.15 Sodium dodecyl sulfate–polyacrylamide gel electrophoresis (SDS‐PAGE)

The rALRase was electrophoresed using Laemmli method (Laemmli, 1970). After rALRase was mixed with sample buffer (EzAppl, AE‐1430; Atto Corp.), it was heated in boiling water for 5 min. The running gel used e‐PAGEL 12.5% (#E‐T12.5L; Atto Corp.). The protein was run at 20 mA for 75 min. After it was gel dyed using Ez Stain Aqua (#AE‐1340; Atto Corp.) standard marker, SDS‐PAGE Molecular Weight Standards (Broad Range, #161‐0317) was used for electrophoresis. *Escherichia coli* BL21 competent cells were used to prepare negative control extraction.

### 2.16 Optimum pH and temperature of the rALRase

Buffers used for this study were 50 mM phosphate–citrate buffer (pH 3.0–6.0), 50 mM phosphate buffer (pH 6.0–8.0), 50 mM CHES‐NaOH (pH 8.0–10.0), and 50 mM phosphate–NaOH buffer (pH 10.0–11.0) for optimum pH of the rALRase. After rALRase (50 μL) was added to 850 μL of each buffer containing 10 mM pyridoxal phosphate (PLP) and 10 mM l‐alanine, the mixtures were incubated at 40°C for 10 min. After the reaction, d‐alanine produced in each pH solution mixture was assayed using d‐amino acid oxidase. For optimum temperature, the rALRase (50 μL) was added to 850 μL of 50 mM CHES‐NaOH (pH 9.0) containing 10 mM PLP and 10 mM l‐alanine. The mixtures were then incubated at 4–80°C for 10 min. Blank 1 and Blank 2 were assayed to calculate ΔA_340_ under each pH or each temperature condition according to the described method. Furthermore, each produced d‐alanine was assayed.

### 2.17 rALRase pH and temperature stability

The rALRase (50 μL) was mixed with 50 μL of each buffer as 50 mM phosphate–citrate buffer (pH 3.0–6.0), 50 mM phosphate buffer (pH 6.0–8.0), 50 mM CHES‐NaOH (pH 8.0–10.0), and 50 mM phosphate‐NaOH buffer (pH 10.0–11.0) at 4°C for 16 h for pH stability of rALRase. The enzyme solution at pH 9.0 (100 μL) was left to stand at 4–80°C for 3 h.

### 2.18 Metal ion and inhibitor effects against rALRase activity

Metal ions (sodium chloride, potassium chloride, magnesium sulfate, zinc sulfate, copper sulfate, cobalt sulfate, iron dichloride, calcium chloride, mercury chloride, and manganese sulfate) were dissolved to a final concentration of 1 mM in 50 mM CHES‐NaOH (pH 9.0) containing 10 mM PLP and 10 mM l‐alanine. Also, inhibitors including iodoacetamide (#I1149; Sigma‐Aldrich Corp.), EGTA and *O*,*O*′‐Bis‐2‐aminoethylethyleneglycol‐*N*,*N*,*N*′,*N*′‐tetraacetic acid (#348‐01311; Dojindo Laboratories), ethylenediaminetetraacetic acid (EDTA, #6381‐92‐6; Dojindo Laboratories), sodium azide, N‐ethylmaleimide, and n‐bromosuccinimide were dissolved to a final concentration of 1 mM in 50 mM CHES‐NaOH (pH 9.0) containing 10 mM PLP and 10 mM l‐alanine. d‐Alanine in reaction mixtures was diluted 10 times with 50 mM CHES‐NaOH buffer (pH 9.0). Then, all were assayed by the mixture solution (150 μL) containing 0.6 μmol min^−1^ mL^−1^ of d‐amino acid oxidase, 25 μmol min^−1^ mL^−1^ of LDH, 0.2 mM NADH, and 0.1 mM each metal ion and inhibitor in 50 mM phosphate buffer (pH 7.0). After the reaction, the solutions were measured at 340 nm using a microplate reader (Multiskan FC; Thermo Fisher Scientific Inc.). Blank 1 and Blank 2 were assayed similar to ALRase to calculate ΔA_340_. Furthermore, the d‐alanine standard curve was made using d‐alanine solution containing each metal ion (final concentration 0.1 mM) or inhibitor (final concentration 0.1 mM) for exclusion effects of inhibiting DAO or LDH by inhibitor or by metal ions.

### 2.19 Kinetics analysis of rALRase

Kinetics of rALRase as *V*_max_ and *K*_m_ values were calculated using Lineweaver–Burk plotting after assaying ALRase within an l‐alanine solution (0.1–50 mM).

### 2.20 Substrate specificity of ALRase

The d‐amino acids produced by ALRase were assayed using peroxide from d‐amino acid by d‐alanine oxidase using peroxidase (Matsumoto & Kanauchi, 2019). First, for the main sample assay, 50 μL of enzyme was added to 850 μL of 1.0 mM l‐amino acid solution (alanine, arginine, aspartic acid, glutamic acid, histidine, isoleucine, leucine, lysine, methionine, phenylalanine, proline, serine, threonine, tryptophan, tyrosine, and valine) in 50 mM CHES (pH 9.0)‐NaOH buffer containing 10 mM PLP. Then, it was reacted at 37°C for 10 min. After the reaction, 50 μL of 100 mM hydrochloric acid solution was added to the reaction mixture to stop the reaction. It was then left to stand for 15 min at 37°C. After 50 μL of 100 mM sodium hydroxide solution was added to the reaction mixture to neutralize it, the mixture was centrifuged at 20,400 *g* for 5 min at 4°C. The supernatant (50 μL) and 150 μL of the mixture solution, which contains DAO (0.6 μmol min^−1^ mL^−1^), POX (10 μmol min^−1^ mL^−1^), 0.11 mM methyl‐benzothiazolinone hydrazone hydrochloride monohydrate, 0.79 mM dimethylaniline, and 10 mM EDTA in 50 mM phosphate buffer (pH 7.0) were poured to microplates (96 wells). Then, they were incubated at 37°C for 30 min. The microplate absorbance was measured at 590 nm using a microplate reader. All standard curves for 0–1.0 mM amino acids were produced to show their activity.

## 3 RESULTS AND DISCUSSION

### 3.1 Cloning of alanine racemase (ALRase)

For this study, the primers for PCR amplification of *alr* were prepared from genomic information in *Lactobacillus sakei* with complete genome sequencing from the National Center for Biotechnology Information (NCBI, 2022). After amplification by PCR, the *alr* was analyzed using pMD20 T‐Vector for TA cloning. Consequently, the amplification of DNA containing coding *alr* was found on the gel for approximately 1300 bp (Data not shown). Then, T‐vector pMD20 and *alr* were ligated. After running electrophoresis, the bands of the DNA ligated T‐vector pMD20 and *alr* (*Escherichia coli* DH5α‐pMD20‐*alr*) were apparent at approx. 4000 bp on agarose gel (data not shown), indicating transgenesis ligating *alr* and pMD20.

*Escherichia coli* DH5α without transformation as negative control had no ALRase activity. On the other hand, *Escherichia coli* DH5α‐pMD20‐*alr* has 12.1 (μmol min^−1^ mL^−1^) ALRase activity (data not shown). Xue et al. (2013) reported that the competent *Escherichia coli* did not find ALRase. Their data agreed with those of earlier studies. Furthermore, Hols et al. (1997) reported that no ALRase‐deleting gram‐positive bacteria strain can be grown without d‐alanine medium because it does not produce cell walls, which are necessary components for d‐amino acids. Palumbo et al. (2004) reported that *L. plantarum*‐deleted ALRase grew normally in d‐alanine‐containing MRS medium. However, in a medium lacking d‐alanine, *L. plantarum* grew and formed abnormal shapes or boring cell walls. However, gram‐negative *Escherichia coli* had small amounts of d‐alanine or d‐amino acid in cells (Miyamoto et al., 2010).

**FIGURE 1:**
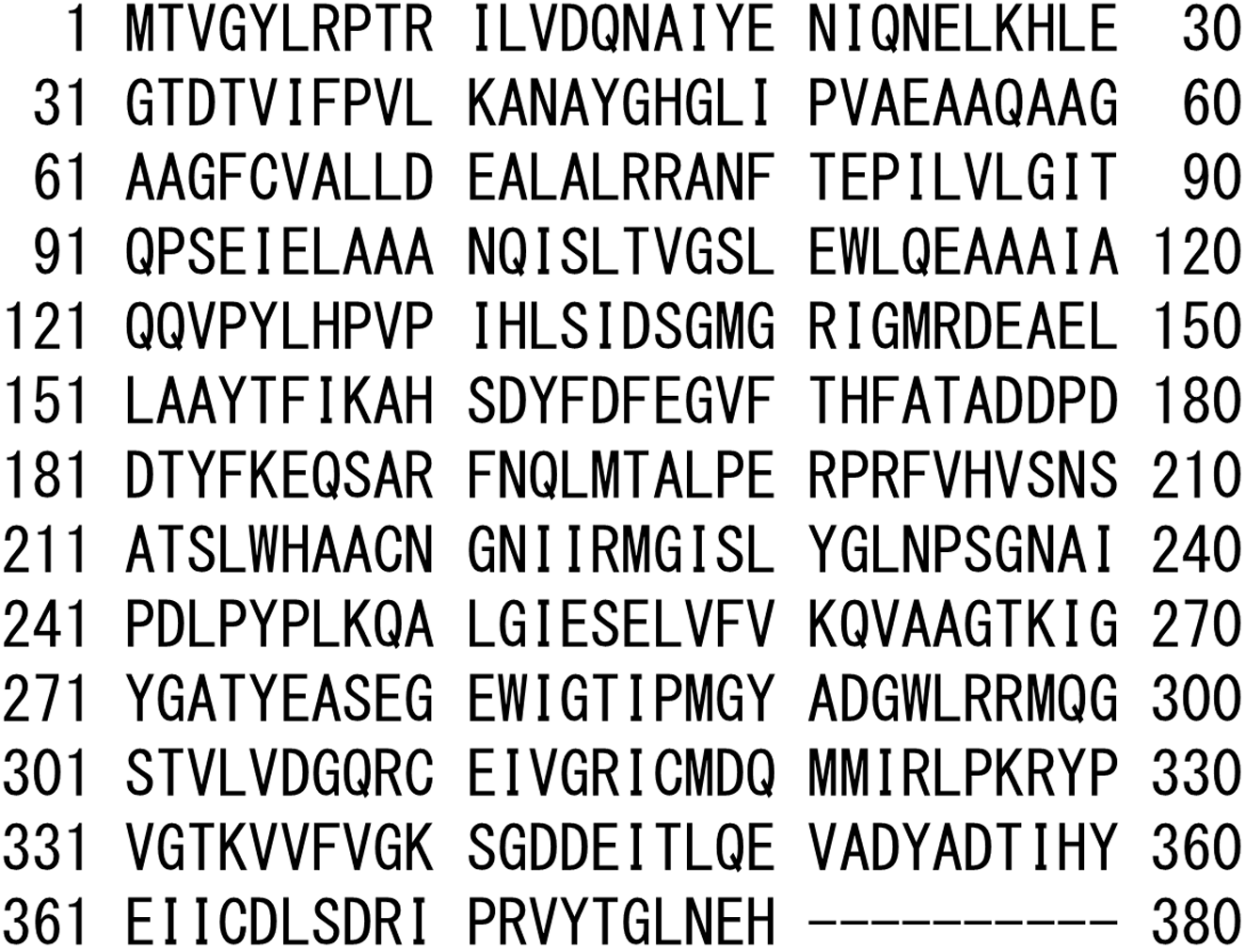
Amino acid sequence of ALRase of ZH2 strain.

Therefore, *Escherichia coli* strain, as the negative control, was not found to have ALRase activity. We inferred that the competent cell was transformed by transgenesis of *alr*. The *alr* was 1140 bp of DNA (data not shown). Its open reading frames (ORFs) have 380 amino acids from the start codon as methionine to that before the termination codon. The ALRase was 41 kDa. The protein had 54.7% hydrophobic amino acids. The relevant data are presented in Figure 1.

The ZH‐2 strain produced a large amount of d‐alanine in the medium. For that reason, it was hypothesized that a*lr* in ZH‐2 strain differs from that of other *Lactobacillus sakei* strains. However, their amino acid sequence in ZH‐2 strain was found to have homology of 99.4% similarity with the *alr* in *Lactobacillus sakei* 23K strain, according to NCBI (http://www.ncbi.nlm.nih.gov/Blast.cgi; Sequence ID: WP_011375306.1; data not shown). At the amino acid sequence, the 117th amino acid in *alr*, threonine in *Lactobacillus sakei* 23K strain, was replaced by alanine in the ZH‐2 strain. It is considered that ‘TTATTTT’ is a Promoter residue found 16 bp upstream from the initiator codon (data not shown). For several reasons, the ZH‐2 strain produces large amounts of d‐alanine in the medium. For example, ZH‐2 strain expressed high‐level ALRase or some other. There might be racemase in ZH‐2 cells that is not this ALRase. *Lactobacillus sakei* had bifunctional amino‐acid racemase with multiple substrate specificities: MalY (Kato & Oikawa, 2018). Future studies must be conducted to analyze other racemization enzymes and ALRase expression levels using rtPCR and its secretion levels.

**FIGURE 2:**
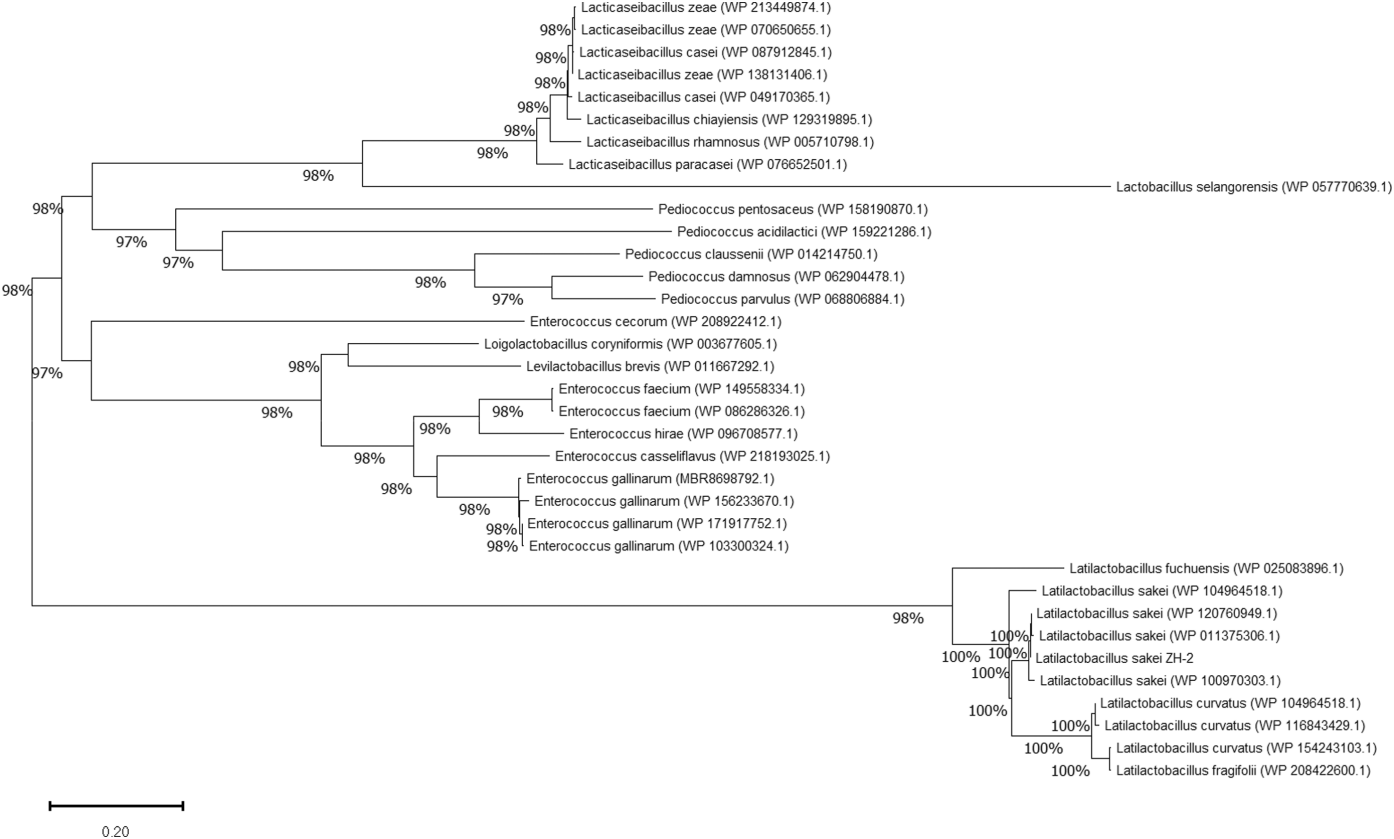
Phylogenetic tree analysis of alanine racemase.

Alanine racemase findings related to phylogenetic tree analysis are presented in Figure 2. To analyze alanine racemase in *L. sakei* ZH‐2 strain by BLAST of homology analysis, amino acid alignment of alanine racemase from 30 strains selected from 250 sequencing data using BLAST (http://www.ncbi.nlm.nih.gov/Blast.cgi) was analyzed using neighbor‐joining method. According to BLAST analysis, alanine racemase in some strains of *L. sakei* had variety of homology of 97–100%. Furthermore, *Latilactobacillus curvatus* had 85%–76% homology, although less than 51% was found for *Enterococcus* with *L. sakei* ZH‐2 strain (data not shown). For phylogenetic tree analysis, their clusters have reliability because all bootstrap values were greater than 94%. It is readily apparent that alanine racemase of *L. sakei* strains had some variety and that the *L. sakei* group and *L. curvatus* group were very close groups according to the phylogenetic tree. By contrast, *Lactobacillus selangorensis*, which was of the same *Lactobacillus* genus, was found to have a weak relation. *Lacticaseibacillus* which belongs to the *Lactobacillus* genus as *L. casei* or *L. paracasei* was also found to have a weak relation. Results suggest that alanine racemase in *L. sakei* ZH‐2 was unique not only in the same *L sakei* group but also in other lactic acid bacteria groups.

Many bacteria have racemases such as ALRase and glutamic acid. PLP, a co‐factor for ALRase, forms a Schiff base in an active center site on PLP‐dependent enzyme as ALRase, as reported by Hayashi (1995). Furthermore, Watanabe et al. (1999) reported that an active site in ALRase of *Bacillus stearothermophilus* had lysine in a 39‐residue amino acid. Some ALRases in *Bacillus* sp. had the amino acid present in the activity center (Shaw et al., 1997; Tanizawa et al., 1988).

In fact, four amino acids have been reported as being in the active center in ALRase *Bacillus* spp. and *Geobacillus* spp.: 129th lysine in amino acid residue (K129), 138th arginine in amino acid residue (R138), 314th methionine in amino acid residue (M314), and 315th aspartic acid in amino acid residue (D315) (Kanodia et al., 2009; Kobayashi et al., 2015; Morollo et al., 1999). Kobayashi et al. (2015) reported histidine and leucine in approximately the 130th amino acid residue as H129 and L130 in ALRmase in *Lactobacillus salivarius*, and H127 and L128 in that of the *Bacillus* spp. and *Geobacillus* sp. H129 and L130. Histidine and leucine (approximately the 130th amino acid residue) are conserved in many bacteria. Furthermore, lysine (K134) or alanine (A131) was located next to the histidine–leucine residue (132–133rd residue or 129–130th residue) in *Lactobacillus*, *Bacillus*, and *Geobacillus* sp. However, ALRase in the ZH‐2 strain, the lysine or alanine next to histidine–leucine at approximately the 130th amino acid residue, had neither. Serine S134 is located at the next H132 and L133 in the enzyme. The 134th amino acid in the ZH‐2 strain replaced serine (S134) from lysine or alanine. However, lysine residue and PLP bind to form a Schiff base. Therefore, some amino acids such as K158, R141, M318, and D319 are thought to have some relation with racemase activity. Future studies will be conducted to investigate amino acid residues at the active center site.

### 3.2 Characteristics of rALRase

**FIGURE 3:**
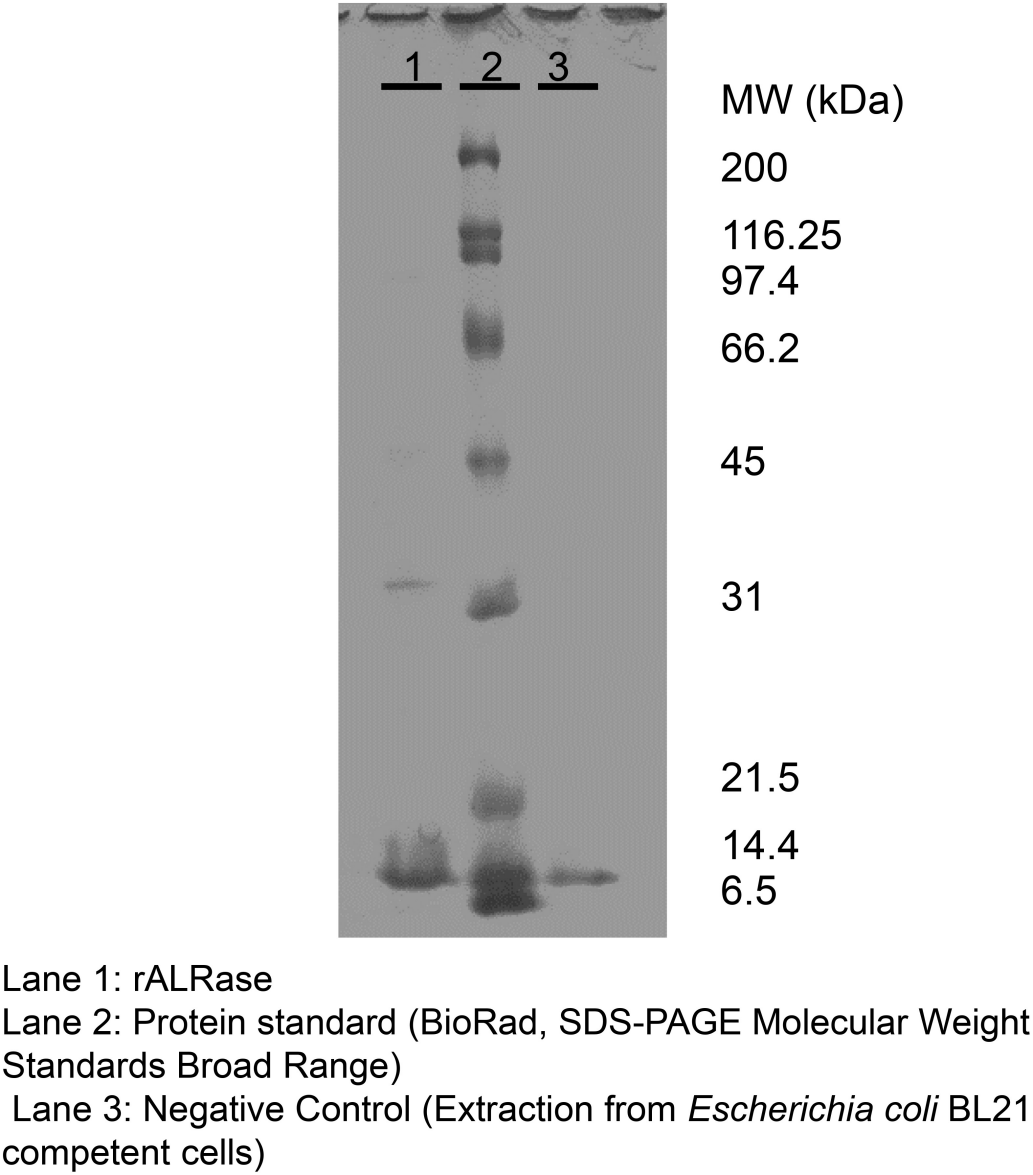
SDS‐polyacrylamide gel electrophoresis (SDS‐PAGE) of rALRase from *Escherichia coli* BL21‐pCold‐*alr*.

Next, pCold‐*alr* ligated *alr* coding ALRase was transferred to *Escherichia coli* BL21 for high expression of ALRase. Its molecular weight according to SDS‐PAGE was found to be 35.7–38.7 kDa (Figure 3), which is less than that of the amino acid sequence (Figure 1). Seow et al. (2000) reported the ALRase of *Thermus thermophilus* as having 38 kDa molecular mass. Kobayashi et al. (2015) reported ALRase of *L. salivarius* as approx. 41 kDa. Their data agreed with molecular mass data of ALR from this amino acid sequence.

**FIGURE 4:**
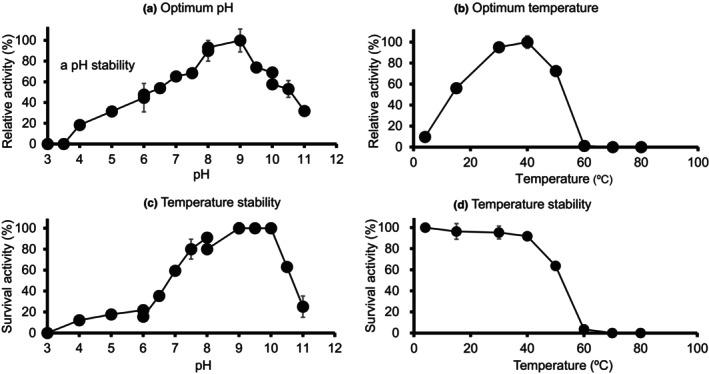
Optimum pH and temperature pH and temperature stability of rALRase from *Escherichia coli* BL21‐pCold‐*alr*.

Next, optimum pH and temperature stability of purified rALRase are presented in Figure 4a,b. Actually, rALRase had the highest activity, at pH 9.0. The highest condition was calculated as having 100% as the relative activity. The rALRase was 90% of the relative activity at pH 8.0, 74% of the relative activity at pH 9.5, and 57%–69% of the relative activity at pH 10. Furthermore, in acid pH ranges, it was 44%–47% of the relative activity at pH 6.0 and 32% of the relative activity at pH 5.0. Results demonstrate that the enzyme reacted more in an alkaline condition than in an acid condition.

Lactic acid bacteria are grown under acidic conditions. But intercellular pH of some bacteria generally keeps at 7.5–8.0 by cell homeostasis (Booth, 1985). And some intercellular enzymes had optimum pH at alkaline condition (Hutkins & Nannen, 1993). In particular, X‐prolyl‐dipeptidyl peptidase in *Lactobacillus sakei* reacted under alkaline condition, and it was approx. 80% of the relative activity at pH 8.0 (Sanz & Toldrá, 2001). Therefore, it is considered that some intercellular enzymes such as rALRase also had optimum pH at alkaline to react in the cytoplasm of the bacteria.

The enzyme reacted at 15–50°C. The highest temperature of 40°C was the optimum temperature of rALRase. In fact, the activity at 40°C was calculated as 100% of the relative activity. Activity at 50°C was calculated as 72% of the relative activity. The activity at 60°C was only 4% of the relative activity. As one might expect, the enzyme activity was nonexistent at temperatures higher than 70°C. Generally, ALRase having lactic acid bacteria was stable in alkaline conditions. For instance, for ALRase in *L. sakei* NBRC15893 isolated from *Kimoto* as *sake* seed mash, Kato and Oikawa (2018) reported optimum conditions of pH 10.0 and 45°C. Moreover, Kobayashi et al. (2015) reported optimum pH for *L. salivarius* with ALRase as 8.0. The optimum conditions of ZH‐2 agree with those for *L. sakei* NBRC15893.

The pH stability and temperature stability of ALRase are presented, respectively, in Figure 4c,d. The enzyme stabilized at pH 9.0–10.0, with decreased activity at pH higher than 10.5: only 63% of the usual activity. The enzyme stabilized when held at 4°C for 16 h. The activity persisted after holding at 4°C: it was calculated to have 100% as the relative activity. The enzyme retained 96% of its activity after holding at 15°C: 92%–95% of the relative activity was retained after holding at 30–40°C. However, only 3% of the relative activity remained after holding at 60°C. Results indicate that the rALRase is stable at 4–40°C. In addition, *L. salivarius* with ALRase had heat tolerance for 30 min at 50°C: 70% of its activity was retained, as reported by Kobayashi et al. (2015). Furthermore, ALRase having *Bacillus anthracis* had heat tolerance, even at 60°C, at which 60% of its activity remained (Kanodia et al., 2009). These results indicate that rALRase has no heat tolerance.

### 3.3 Effects of rALRase against metal ions and inhibitors

Effects of rALRase against metal ions and inhibitors are presented in Table 1. For this enzyme assay method, ALRase, amino acid oxidase, and lactic acid dehydrogenase were used. Therefore, to assay rALRase within ions or inhibitors precisely, a standard curve of d‐alanine was produced using the d‐alanine solution, oxidase, and lactic acid dehydrogenase within each metal ion or inhibitor. The rALRase was assayed and compared with and without PLP in the reaction mixture system. The rALRase with PLP in the reaction mixture had 1.5–2 times the activity of the reaction mixture without PLP (data not shown). It is readily apparent that its cofactor was PLP.

**TABLE 1:** Metal and inhibitor solution effects.

Metal/inhibitor	Relative activity (%)
Metal ion (1 mM)	
CaCl_2_	90.8 (±1.68)^a^
CoSO_4_	59.6 (±0.42)^a^
CuSO_4_	133.8 (±1.16)^b^
FeCl_2_	80.8 (±3.16)^ac^
MgSO_4_	62.4 (±0.89)^ac^
MnSO_4_	79.7 (±7.32)^ac^
HgCl_2_	25.9 (±9.31)^ac^
KCl	87.3 (±4.192)^ac^
NaCl	88.8 (±4.64)^ac^
ZnSO_4_	327.4 (±1.03)^d^
Inhibitor (1 mM)	
Azid	75.2 (±5.64)^ace^
BSF	59.4 (±0.79)^ace^
EDTA	59.6 (±0.21)^ace^
EGTA	55.6 (±2.85)^ace^
Iodoacetamide	65.7 (±8.33)^ace^
NEM	62.7 (±3.59)^ace^
NBS	67.7 (±4.69)^ace^
Control	100.0 (±0.21)^abce^

*Note*: Tukey's multiple comparisons of mean, *F*_(17,36)_ = 599.62, *p* < .05.

Different letters show statistical significance.

Generally, enzymes of many kinds have an active center site combining metal ions, which allow for effective enzymic catalysis. Standard curves of d‐alanine using solutions containing each metal ion (final concentration 0.1 mM) or inhibitor (final concentration 0.1 mM) and the enzyme system using two enzymes as DAO and LDH might be inhibited by some metal ions or inhibitors. Consequently, the standard curve of d‐alanine was produced under existing metal ion or inhibitor conditions. Their metal and inhibitor concentrations were regarded as being of low levels, such as 0.1 mol/L (data not shown), after rALRase reaction solutions. Therefore, all standard curves were made without metal ion or inhibitor inhibition of DAO or LDH. Their relative activity (100%) was recorded as the activity without the metal ion or inhibitor (Table 1). The enzyme activity was activated by copper and zinc ions. It was 133% of the relative activity by copper ion and 327% of the relative activity by zinc ion. It was significantly highest activity.

One kind of PLP‐dependent enzyme was activated by the metal ion. For instance, Yoshimura and Goto (2008) reported that serine dehydrogenase was activated by zinc ion. Moreover, they described that its ion combined cysteine amino acid residues at its active center sites. Furthermore, some enzymes were deactivated by mercury ion binding with cysteine residue at the active site (Ynalvez et al., 2016). Although rALRase had five cysteine residues in components, only one cysteine, C317, is located by M318 and D319 at the main active site. Probably, mercury ions are deactivated from binding with C317. Moreover, the effects of inhibitors, chelating reagents of EDTA and EGTA, inhibited rALRase, suggesting the respective activities of 59.6% and 55.6%. These findings demonstrate that the enzyme requires metal ions for enzymes.

Yamashita et al. (2003) reported ALRase of *Bifidobacterium* spp. as inhibited by DTNB, which reacts with sulfhydryl group in amino acid residues (Ajsuvakova et al., 2020; Bamforth et al., 2009). The ALRase was inhibited by divalent ions as a mercury ion sulfhydryl group in amino acid residues. Both enzymes are regarded as having a sulfhydryl group in amino acid residues in the active center.

Actually, PLP was a co‐factor of this ALRase (Hayashi, 1995). The rALR had lysine, arginine, methionine, and aspartic acid in the active center, but it had no cysteine there. These findings agree with those of reports describing that PLP‐dependent enzymes are classifiable into five groups of ALRase produced from ALRase‐classified fold‐III type, which is a necessary divalent ion (Knight et al., 2017; Steffen‐Munsberg et al., 2015; Tanaka et al., 2011).

### 3.4 Kinetic analyses and kinetic analyses of rALRase

**FIGURE 5:**
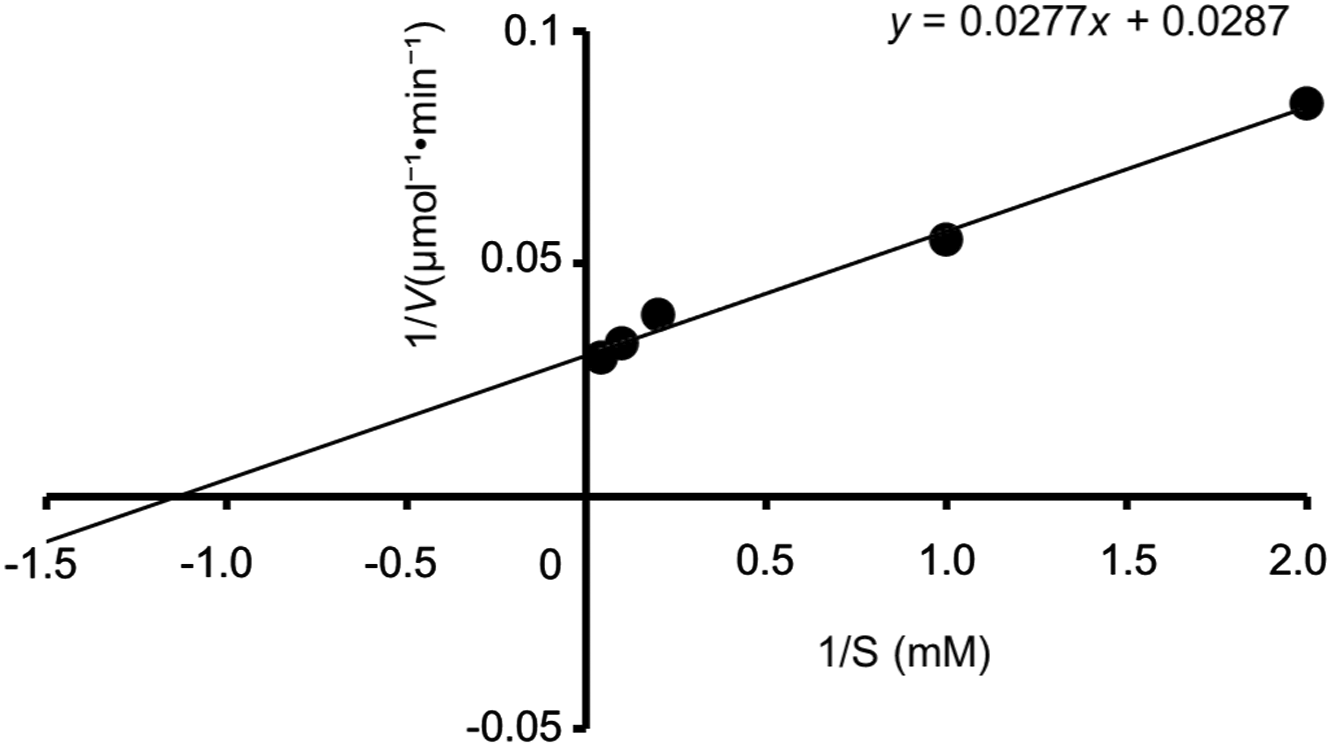
Lineweaver–Burk plot of rALRase activity from *Escherichia coli* BL21‐pCold‐*alr*.

Results of kinetic analyses of rALRase are presented in Figure 5. After rALRase was assayed against each concentration of alanine solution, the data and alanine concentrations were presented as a Lineweaver–Burk plot. There, *K*_m_, representing the affinity of the substrate and enzyme, was 1.32 × 10^−3^ (M). In addition, *V*_max_, signifying the reaction speed, was 4.27 × 10^−5^ (μmol^−1^ min^−1^). ALRase having *L. salivarius* was 5.33 × 10^−3^ (M), demonstrating that the ALRase from ZH‐2 has low *K*_m_. It had high‐affinity characteristics with substrates such as l‐alanine.

Substrate specificity of rALR was analyzed. ALRase assay specifically decreased NADH by lactic acid dehydrogenase and pyruvic acid produced from d‐alanine by d‐alanine oxidase. Using this method, ALRase was not useful for assay against other substrates. Therefore, ALRase for other substrates was assayed with peroxide from d‐amino acid by d‐alanine oxidase using peroxidase according to the method reported by Matsumoto and Kanauchi (2019). That earlier report described that the crude ALRase from ZH‐2 strain reacted most against l‐alanine in other amino acids. Moreover, rALRase reacted most against l‐alanine in other amino acids.

**FIGURE 6:**
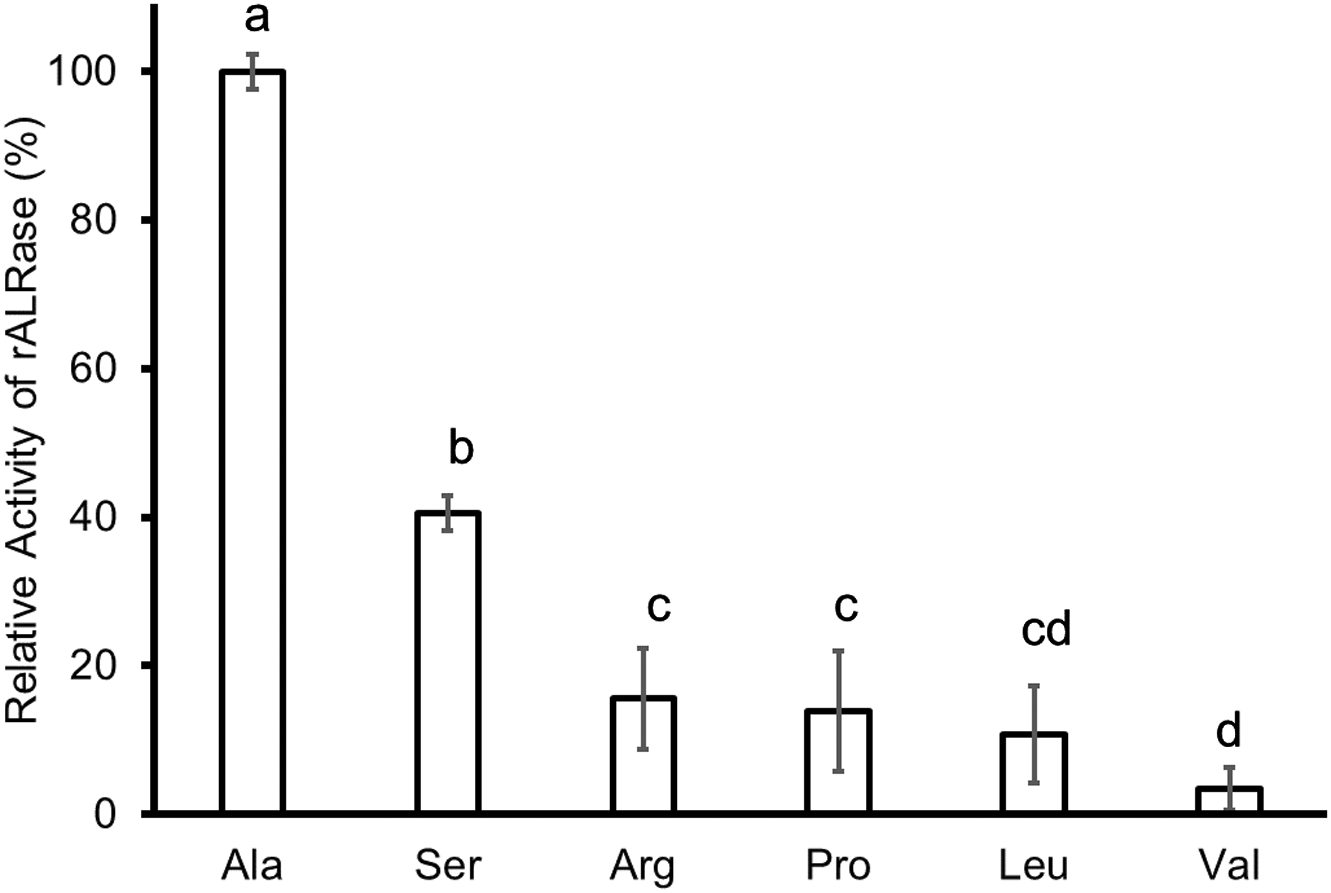
Substrate specificity of rALRase from *Escherichia coli* BL21‐pCold‐*alr*. Tukey's multiple comparisons of mean, *F*_(5,12)_ = 1863.9, *p* < .05.

The activity of rALRase (13.8 μmol min^−1^ mL^−1^) was calculated as 100% of the relative activity. The data are portrayed in Figure 6. The enzyme reacted against serine was 40% of the relative activity. The enzyme reacted against arginine was 15%. The enzyme reacted against proline was 14%. The enzyme reacted against leucine was 10% because d‐alanine conversion by ALRase included the peptidoglycan layer in the cell wall (Hols et al., 1997). d‐Alanine and d‐glutamic acid included all four classified peptidoglycan types. *Lactobacillus sakei* has A4β type peptidoglycan composing d‐alanine, d‐glutamic acid, and d‐aspartic acid (Lund et al., 1999). However, this racemase did not react against aspartic acid or glutamic acid. Kato and Oikawa (2018) reported that amino acid racemase in *Lactobacillus sakei* has activity against glutamic acid, but it has very low activity. Furthermore, they reported that alanine is reacted most by the enzyme in others. These data agree with those presented in their report.

Yoshimura and Goto (2008) reported that serine racemase in the brain has 31% similar homology to ALRase of *B. stearothermophilus*. They also reported that important residues are conserved for the catalysis racemase reaction. Therefore, we infer that ALRase in ZH‐2 strain also has activity against serine and other amino acids. However, this ALRase has no activity against histidine, isoleucine, lysine, methionine, phenylalanine, threonine, tryptophan, or tyrosine.

### 3.5 Substrate–product equilibrium of rALRase

**FIGURE 7:**
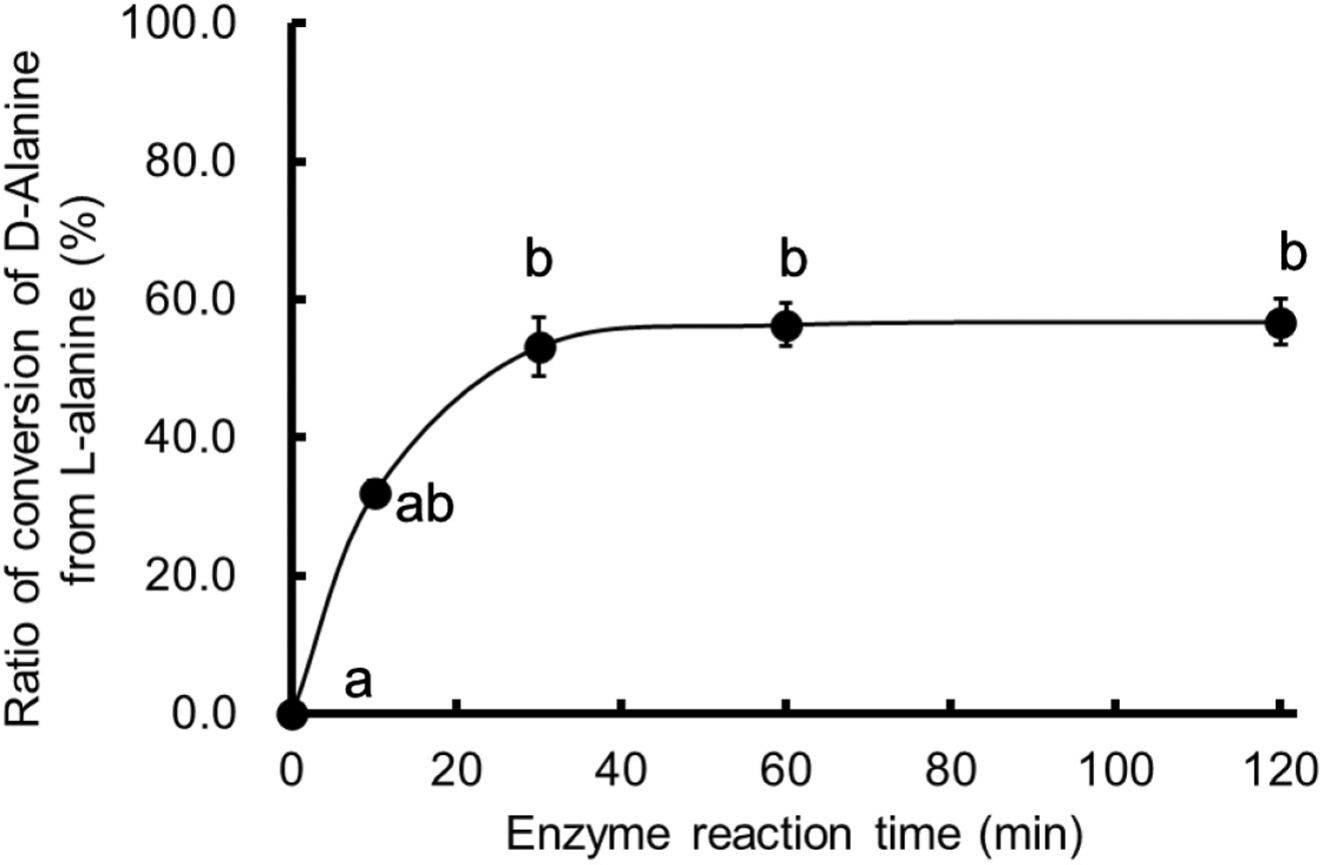
Ratio of conversion of d‐alanine from l‐alanine by rALRase from *Escherichia coli* BL21‐pCold‐*alr*. Tukey's multiple comparisons of mean, *F*_(4,10)_ = 33.14, *p* < .05.

Finally, the substrate–product equilibrium of rALRase was analyzed to assay d‐alanine (Figure 7). The enzyme converted d‐alanine from l‐alanine quickly for up to 10 min. Then, it converted it slowly after 10 min. For more than 30 min, the conversion ratio of d‐alanine from l‐alanine was 56.0%. ALRase in *Aeromonas hydrophila* was keq(L/D)1.0 (Liu et al., 2015). The enzyme will convert 50% of the substrate: until l‐alanine:d‐alanine (1:1). Results show that *Lactobacillus sakei* ZH‐2 strain has some potential to produce d‐amino acid as d‐alanine for the production of good *sake*. For this study, this rALRase was not assayed to convert l‐alanine from d‐alanine. Assay of *K*_m_ of racemase against conversion of d‐alanine to l‐alanine will be undertaken in future studies.

## 4 CONCLUSION

The *Lactobacillus sakei* ZH‐2 strain with alanine racemase (ALRase) was analyzed for application to food production. The ALRase was expressed using transformed *Escherichia coli* DH5α‐pMD20‐*alr* strain. The transduction *Escherichia coli* DH5α‐pMD20‐*alr* had 13.9 (μmol^−1^ min^−1^). The *alr* DNA was 1140 bp; ALRase has 41 kDa molecular weight. However, its molecular weight was found to be 35.7–38.7 kDa by SDS‐PAGE. Results showed 99.4% similarity of homology of amino acid sequence with complete genome‐sequenced *Lactobacillus sakei* 23K. For high expression of ALRase, the transformed strain was designated as *Escherichia coli* BL21‐pCold‐*alr* strain. Consequently, expressing ALRase was obtained. Its optimum pH is 9.0, but results indicate that it is stable at pH 9.0 and pH 10.0. Its optimum temperature is 30–40°C, but it is stable at 4–40°C. Heating to temperatures higher than 60°C eliminates its activity. Its co‐factor was found to be PLP. It is activated by copper ion and zinc ions such as bivalent ions. This PLP‐dependent and metal‐requiring enzyme has *K*_m_ of 1.32 × 10^−3^ (mol). Its *V*_max_ is 4.27 × 10^−5^ (μmol^−1^ min^−1^). Results obtained from this study demonstrate that this racemase has higher affinity with the substrate than others. Substrate specificity of the ALRase was assayed using d‐amino acid oxidase, which can assay many d‐amino acids. Alanine was reacted most by rALRase in other substrates such as amino acids. This rALRase also reacted weakly with serine, arginine, and proline. The enzyme converted 54.5% of d‐alanine from l‐alanine while demonstrating potential to produce d‐amino acid as d‐alanine.

## AUTHOR CONTRIBUTIONS

**Makoto Kanauchi:** Conceptualization (equal); data curation (equal); formal analysis (equal); funding acquisition (equal); investigation (equal); methodology (lead); project administration (lead); resources (lead); software (lead); supervision (lead); validation (lead); visualization (lead); writing – original draft (lead); writing – review and editing (lead). **Natsuki Matsumoto:** Conceptualization (equal); data curation (equal); formal analysis (equal); funding acquisition (equal); investigation (equal); methodology (supporting); project administration (supporting); resources (supporting); software (supporting); supervision (supporting); validation (supporting); visualization (supporting); writing – original draft (supporting); writing – review and editing (supporting).

## CONFLICT OF INTEREST STATEMENT

The authors have no conflict of interest.

## Data Availability

The data that support the findings of this study are available from the corresponding author upon reasonable request.
